# Evaluating organ delineation, dose calculation and daily localization in an open-MRI simulation workflow for prostate cancer patients

**DOI:** 10.1186/s13014-014-0309-0

**Published:** 2015-02-11

**Authors:** Anthony Doemer, Indrin J Chetty, Carri Glide-Hurst, Teamour Nurushev, David Hearshen, Milan Pantelic, Melanie Traughber, Joshua Kim, Kenneth Levin, Mohamed A Elshaikh, Eleanor Walker, Benjamin Movsas

**Affiliations:** Department of Radiation Oncology, Henry Ford Health System, 2799 W. Grand Blvd, Detroit, MI 48202 USA; Department of Radiation Oncology, 21st Century Oncology, 28585 Orchard Lake Rd, Suite 110, Farmington Hills, MI 48334 USA; Department of Radiology, Henry Ford Health System, 2799 W. Grand Blvd, Detroit, MI 48202 USA; Philips Healthcare, 603 Alpha Park, Cleveland, OH 44143 USA

**Keywords:** MRI simulation, CBCT localization, MRI dose calculation, Anatomical delineation, Radiation Oncology

## Abstract

**Background:**

This study describes initial testing and evaluation of a vertical-field open Magnetic Resonance Imaging (MRI) scanner for the purpose of simulation in radiation therapy for prostate cancer. We have evaluated the clinical workflow of using open MRI as a sole modality for simulation and planning. Relevant results related to MRI alignment (vs. CT) reference dataset with Cone-Beam CT (CBCT) for daily localization are presented.

**Methods:**

Ten patients participated in an IRB approved study utilizing MRI along with CT simulation with the intent of evaluating the MRI-simulation process. Differences in prostate gland volume, seminal vesicles, and penile bulb were assessed with MRI and compared to CT. To evaluate dose calculation accuracy, bulk-density-assignments were mapped onto respective MRI datasets and treated IMRT plans were re-calculated. For image localization purposes, 400 CBCTs were re-evaluated with MRI as the reference dataset and daily shifts compared against CBCT-to-CT registration. Planning margins based on MRI/CBCT shifts were computed using the van Herk formalism.

**Results:**

Significant organ contour differences were noted between MRI and CT. Prostate volumes were on average 39.7% (p = 0.002) larger on CT than MRI. No significant difference was found in seminal vesicle volumes (p = 0.454). Penile bulb volumes were 61.1% higher on CT, without statistical significance (p = 0.074). MRI-based dose calculations with assigned bulk densities produced agreement within 1% with heterogeneity corrected CT calculations. The differences in shift positions for the cohort between CBCT-to-CT registration and CBCT-to-MRI registration are −0.15 ± 0.25 cm (anterior-posterior), 0.05 ± 0.19 cm (superior-inferior), and −0.01 ± 0.14 cm (left-right).

**Conclusions:**

This study confirms the potential of using an open-field MRI scanner as primary imaging modality for prostate cancer treatment planning simulation, dose calculations and daily image localization.

## Background

Magnetic resonance imaging (MRI) is a supplement to computed tomography (CT) in radiation oncology where superior soft tissue contrast is required for anatomical delineation. MRI simulation in radiation therapy (RT) could potentially be a routine modality in the future primarily because MRI offers the ability to image tumors and surrounding healthy tissues with significantly better soft tissue contrast than CT (lung being a notable exception). MRI is an established standard for target and organ-at-risk delineation for brain and spinal cord cancers and is used in conjunction with CT for treatment planning [1-3]. In prostate treatment in particular, MRI as a visualization tool for structures has been investigated [4-7].

MRI-only workflows have been introduced with the primary endpoint of eliminating the need for CT simulation (CT-SIM). Devic [8] provides an excellent review of the current use of MRI in radiation oncology, including MRI simulation. Recently, MRI-only simulators have been introduced, incorporating features commonly found in CT-SIM: flat table tops to accommodate immobilization devices and external laser systems for patient marking and alignment. These two major additions facilitate better agreement in patient positioning between MRI and radiation therapy delivery. As MRI technology continues to evolve, new radiation therapy systems are utilizing MRI as an on-board localization technology, such as the three cobalt source unit from ViewRay (Oakwood Village, OH) [9], the MRI-LINAC from Elekta (Stockholm, SE) [10], or the MRI-on-rails solution being installed at Princess Margaret Hospital in Toronto, CA [11].

Nevertheless, CT-SIM remains the gold standard for treatment planning because CT voxels demonstrate a tissue’s electron density, a factor critical to determining the absorbed dose, as well excellent geometric accuracy and less stringent safety considerations. Electron density information is not easily accessible from MRI; and in an MRI-only simulation workflow treatment planners need to assign bulk densities to multiple contours that are not currently generated in a CT workflow. MRI treatment planning is currently a topic of considerable interest in radiation oncology [12-15]. Despite its promise, issues related to geometric distortions (stemming from magnetic field inhomogeneities [16,17] and patient-induced artifacts [18,19]), dose calculation [20-22] and the use of MRI datasets for image-guidance need to be optimized to facilitate accurate and efficient clinical workflow.

This study’s goal was to evaluate the potential of vertical-field open design MRI scanner as a primary modality for simulation, planning, and IGRT-based localization for treatment of patients with prostate cancers. Dose calculation accuracy was evaluated, including the use of bulk density assignments to overcome limitations in electron density conversion of the MRI signal. A unique aspect of our study centers on using MRI as a reference dataset for CBCT-MRI, 3D-3D image registration for image-guided RT. Such data is important for routine clinical use of MRI, and has not been previously published. Additionally, for an open platform MRI simulator, we investigated issues related to image acquisition sequences, and the possible pitfalls of improper imaging and its effect on anatomical contouring.

## Methods

**Figure 1:**
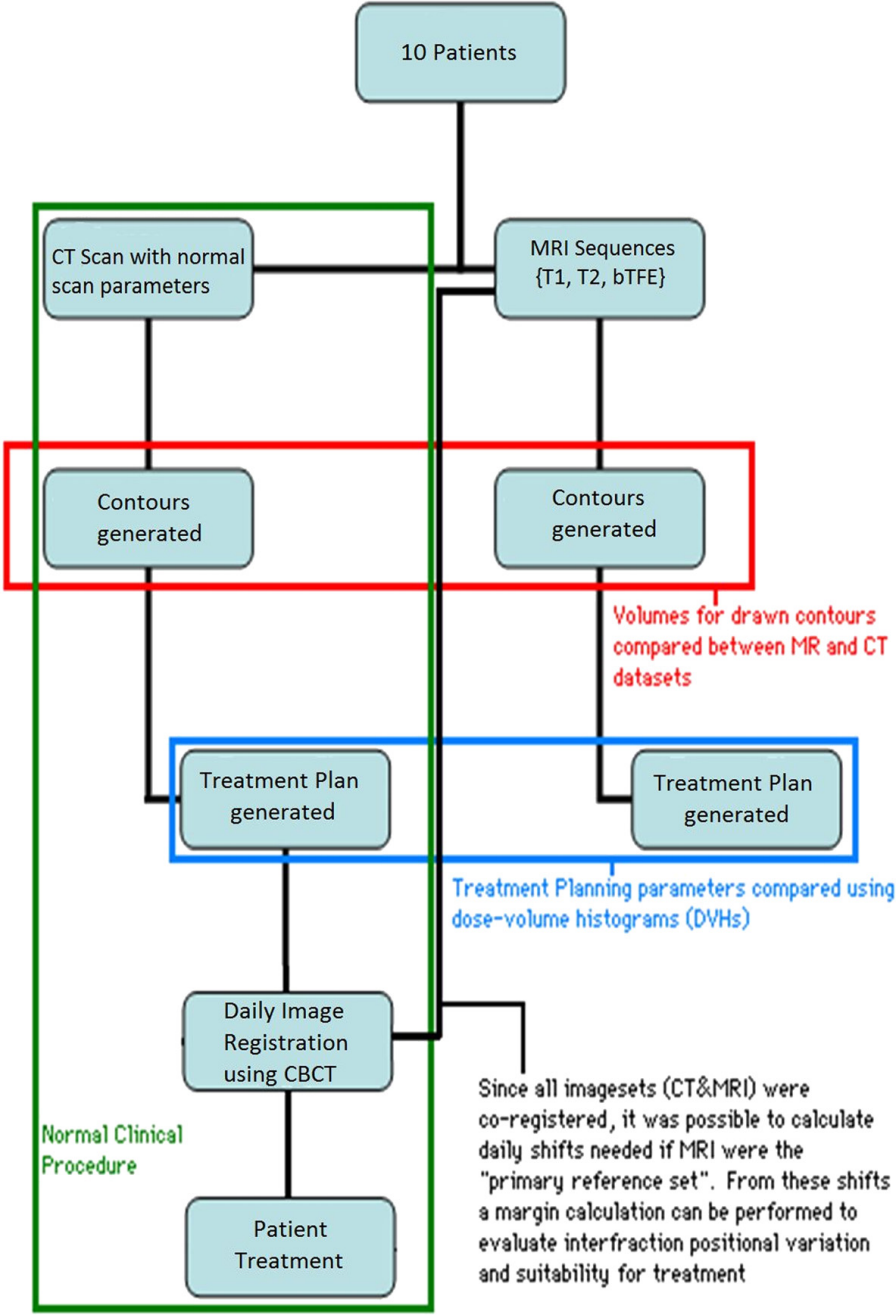
**Study workflow.**

Ten prostate cancer patients enrolled on an Institutional Review Board approved study (to be in compliance of the Helsinki declaration) had a MRI acquired along with standard CT simulation. MRIs were obtained from a one Tesla (1.0 T) open MRI scanner (Panorama, Philips Healthcare, Best, Netherlands) that has been developed as a simulation device for RT procedures. Figure 1 shows our workflow for CT and MRI simulation. Whole pelvis acquisitions were performed using T1-weighted Fast Field Echo, T2-weighted Turbo Spin Echo and 3D balanced Turbo Field Echo (bTFE) sequences. Table 1 shows acquisition parameters for these scans. T1 and T2 scans were full field of view (FOV) in order to use them for treatment planning. The bTFE scans were ‘coned-down’ to smaller regions of interest to help keep acquisition time down and allow for improved organ delineation. Of note is that these acquisition parameters are optimized for RT planning by the manufacturer, and are not applicable to diagnostic imaging protocols. A flat table top insert (Civco, Orange City, IA) was used to reproduce typical patient positioning.

**Table 1:** **MRI acquisition parameters**

	**T2 TSE**	**T1 FFE**	**bTFE**
**Weighting**	T2	T1	T2/T1
**Acquisition**	2D	3D	3D
**Scan duration (min:sec)**	07:13.5	07:38.8	06:18.9
**TR (ms)**	4563	17	5.4
**TE (ms)**	80	6.9	2.7
**ACQ voxel (mm)**	1.00/1.00/2.50	1.15/1.50/2.50	1.25/1.25/2.50
**REC voxel (mm)**	0.69/0.69/2.50	0.69/0.69/2.50	0.60/0.60/2.50
**FOV AP (mm)**	300	300	260
**RL (mm)**	440	440	200
**FH (mm)**	225	225	225
**Coil selection**	BodySp-XL	BodySp-XL	BodySp-XL
**Flip angle (deg)**	90	25	75
**Orientation**	axial	axial	axial
**Number of Signal Averages (NSA)**	1	1	2
**Fat Saturation**	none	none	SPIR
**TSE echo spacing/shot (ms)**	8.0 / 200	-	-
**TFE factor**	-	-	256

The MRI simulation process shares many aspects with CT simulation. The anatomy of interest (pelvis, head, etc.) is externally targeted and then moved to the magnet isocenter. A three plane survey (analogous to CT scouts) is performed with the largest FOV to determine the acquisition volume. Acquisition time for three whole pelvis sequences totals twenty minutes.

### Anatomical contouring

All anatomical delineation and planning were done in Eclipse® (Varian Medical Systems, Palo Alto, CA). First, all MRIs were rigidly registered to CT images focusing on a match of the prostate. Secondly, organ delineation was performed/reviewed by a single observer for the T1-MRI and CT images. Retrospectively, another observer contoured the T2 and bTFE volumes. All contours were reviewed by an attending physician.

### Treatment planning

All patients were planned using intensity modulated radiation therapy (IMRT) with seven or nine fields. The clinical target volume (CTV) was defined as prostate or as prostate plus proximal seminal vesicles based on disease staging. For patient treatment, the prostate volume was based on the CT volume instead of the MRI drawn prostate volume. The planned target volume (PTV) was an expansion of the CTV by 1 cm in all directions except 0.6 cm posteriorly. IMRT dose constraints followed QUANTEC [23] guidelines. Plans were prescribed so the dose to ninety-five percent of the volume (D95) was 75.6 Gy in 42 fractions.

Retrospective dose calculations were performed on T1-MRI images using bulk-density-assignment for each of the structures (body contour, PTV, seminal vesicles, bladder, rectum, penile bulb and bony structure), by assigning bony tissue to 480 Hounsfield units (HU) and soft tissue to 0 HU (water equivalent). The bone value (480 HU) was determined by contouring bony tissue on CT and measuring the average CT number. Soft tissue is comprised of fat and muscle which have HU values slightly lower and higher than water, respectively, so we chose to represent all soft tissue with water density. No air pockets were present in the treated area. Had they been present, a value of −1000 HU would have been used. Bulk-density-assignment for dose calculation using MRI datasets in the context of prostate cancer have been used by other investigators [13,21]. MRI datasets cannot be used to generate dose plans in Eclipse v11. Instead, unit-density CT datasets were registered to MRI datasets and MRI contours copied to CT to create workable datasets for dose calculation. The same beam angles, leaf segment positions and monitor units were calculated to isocenter. We evaluated the impact of bulk-density-assignment on IMRT optimization using a few representative datasets. Inverse planning was performed using bulk-density-assignment and plans were compared to those in which actual CT densities were used. For both datasets, the identical beam arrangements, plan and optimization parameters were used for inverse planning. The calculated DVHs for the prostate gland, bladder and rectum were compared.

### CBCT-based treatment localization

IMRT delivery was performed on Trilogy® and Truebeam® linear accelerators (Varian Medical Systems, Palo Alto, CA). All patients underwent daily CBCT localization and shifts were recorded. Retrospectively, another observer used MRI datasets as the primary reference sets, allowing for virtual realignment of all CBCT images (~400 datasets). MRI-CBCT registration was performed manually prioritizing alignment of the prostate-rectum interface correctly, which is standard practice. By analyzing these shifts and using the van Herk formalism [16], we calculated the CTV-to-PTV margins needed for the CT and MRI reference sets. This formalism calculates the margins needed to deliver 95% of the prescribed dose to 90% of the patient population. It is calculated using the cohort’s inter-patient uncertainty (Σ) and the inter-fraction uncertainty (σ) in the formula [24].

Margin = 2.5 Σ + 0.7 σ. We also calculated the group’s mean error (M). The inter-patient uncertainty (Σ) is calculated by averaging all daily shifts and then taking the standard deviation of each patient’s average value. The inter-fraction uncertainty (σ) is calculated by taking the standard deviation of all daily shifts and then taking the root mean square (RMS) of each patient’s standard deviation.

## Results

### Anatomical contouring

Volumetric differences in contours from MRI and CT datasets are summarized in Table 2. Significant differences were noted between MRI and CT. Prostate and penile bulb volumes were on average 39.7% (p = 0.002) and 61.1% (p = 0.074) larger on CT than on MRI respectively. The prostate volume difference agrees well with values from Roach et al. [25] Seminal vesicle volumes were underestimated on the CT scan by 1.7% (p = 0.454) compared to the volumes from MRI. CTV (prostate plus seminal vesicles) were on average 27.5% (p = 0.002) smaller on the MRI than they were on CT.

**Table 2:** **Anatomical differences between MRI and CT Datasets**

	**Prostate**		**Seminal Vesicles**		**Penile Bulb**		**CTV (prostate + seminal vesicles)**	
	**MRI vol (cc)**	**CT vol (cc)**	**MRI vol (cc)**	**CT vol (cc)**	**MRI vol (cc)**	**CT vol (cc)**	**MRI vol (cc)**	**CT vol (cc)**
**Patient 1**	79.46	89.63	16.43	19.96	2.63	3.02	95.89	109.59
**Patient 2**	83.70	131.00	18.23	7.66	12.98	12.02	101.93	138.66
**Patient 3**	37.84	56.44	16.22	14.19	3.95	4.45	54.06	70.63
**Patient 4**	84.20	117.60	11.17	16.47	5.25	8.76	95.37	134.07
**Patient 5**	26.81	45.14	6.85	9.02	1.95	4.98	33.66	54.16
**Patient 6**	31.78	50.20	26.59	15.37	4.04	7.15	58.37	65.57
**Patient 7**	38.94	44.46	22.92	17.27	3.64	4.93	61.86	61.73
**Patient 8**	30.82	42.13	8.80	5.24	3.44	0.55	39.62	47.37
**Patient 9**	32.03	42.50	9.44	12.64	2.81	4.74	41.47	55.14
**Patient 10**	31.76	40.83	14.26	18.02	1.58	5.87	46.02	58.85
**%Diff (CT to MRI)**	39.7%		−1.7%		61.1%		27.5%	
**St Dev**	18.5%		38.1%		96.8%		17.1%	
**P value**	0.002		0.454		0.074		0.002	

### Treatment planning

Inverse-optimized plans using either bulk-density-assignment or CT-pixel densities have dose parameters that differ on average by less than 0.5% which was characterized for head and neck IMRT cases by Karotki et al. [21] We analyzed the dose to 99% and 95% of the MRI-simulation drawn prostate (D99) and (D95) along with the global maximum dose for the MRI- and CT-based plans. Patient results are presented in Table 3. Patient eight could not have bulk-density-assignment because a hip prosthetic did not allow for accurate contouring of femoral heads and surrounding bony tissue. The global maximum dose differed by 1.01% between MRI and CT plans for the patient cohort, and both the D99 and D95 were within 0.2% of the CT-based dose calculation.

**Table 3:** **Dose differences between CT-based dose calculation and MRI bulk density assigned dose calculation**

	**Plan Max Dose**		**D99 for Prostate**		**D95 for Prostate**	
	**MRI (Gy)**	**CT (Gy)**	**MRI (Gy)**	**CT (Gy)**	**MRI (Gy)**	**CT (Gy)**
**Patient 1**	76.61	76.97	72.45	73.70	72.65	73.90
**Patient 2**	82.43	79.58	77.58	76.05	77.82	76.30
**Patient 3**	76.17	74.01	70.24	69.78	70.60	70.18
**Patient 4**	74.81	75.17	70.83	69.10	71.15	70.89
**Patient 5**	74.25	75.13	70.54	72.01	70.73	72.09
**Patient 6**	78.02	77.05	73.80	73.76	73.94	73.92
**Patient 7**	78.78	77.46	73.67	73.42	73.81	73.55
**Patient 9**	81.59	79.62	76.77	75.77	76.98	75.95
**Patient 10**	74.97	75.25	70.63	72.03	70.70	72.13
**Avg Dose Diff.**	−1.01%		−0.11%		0.12%	
**STDEV**	1.69%		−0.34%		−0.09%	
**P value**	0.104		0.816		0.873	

### CBCT-based treatment localization

Patient 8 was not evaluated for this section due to metal artifacts impacting CBCT image quality. The averages and standard deviations of daily shifts used for treatment localization for each patient are summarized in Table 4. The differences in shift positions for the entire cohort between CBCT-to-CT registration and CBCT-to-MRI registration were −0.15 ± 0.25 cm in the AP direction, 0.07 ± 0.19 cm in the SI direction and −0.01 ± 0.14 cm in the LR direction.

**Table 4:** **Average and standard deviations values for CBCT shifts for all fractions**

	**CT registered to CBCT**			**MRI registered to CBCT**		
	**AP (cm)**	**SI (cm)**	**LR (cm)**	**AP (cm)**	**SI (cm)**	**LR (cm)**
**Patient 1**	0.21 ± 0.28	0.04 ± 0.25	0.04 ± 0.29	−0.07 ± 0.25	0.11 ± 0.11	0.03 ± 0.27
**Patient 2**	0.49 ± 0.30	−0.02 ± 0.54	0.07 ± 0.36	0.44 ± 0.28	0.03 ± 0.54	0.04 ± 0.33
**Patient 3**	0.04 ± 0.61	0.06 ± 0.53	0.06 ± 0.51	0.01 ± 0.76	0.23 ± .58	0.01 ± 0.48
**Patient 4**	0.10 ± 0.23	0.18 ± 0.24	−0.08 ± 0.26	0.02 ± 0.15	0.02 ± 0.15	0.00 ± 0.00
**Patient 5**	0.17 ± 0.32	0.28 ± 0.22	−0.06 ± 0.36	0.27 ± 0.47	0.42 ± 0.27	−0.15 ± 0.38
**Patient 6**	0.26 ± 0.42	0.02 ± 0.24	0.16 ± 0.19	−0.02 ± 0.33	0.10 ± 0.20	0.10 ± 0.18
**Patient 7**	−0.06 ± 0.49	−0.18 ± 0.88	0.11 ± 0.64	−0.63 ± 0.49	−0.11 ± 0.86	0.15 ± 0.64
**Patient 9**	−0.33 ± 0.28	−0.10 ± 0.16	0.30 ± 0.25	−0.28 ± 0.29	−0.02 ± 0.11	0.29 ± 0.27
**Patient 10**	0.36 ± 0.30	0.05 ± 0.15	−0.32 ± 0.31	0.19 ± 0.29	0.04 ± 0.14	−0.31 ± 0.33
**M**	0.14	0.04	0.03	−0.01	0.09	0.02
**Σ**	0.24	0.14	0.17	0.31	0.15	0.17
**σ**	0.38	0.43	0.38	0.41	0.41	0.36
**Margin**	0.87	0.64	0.69	1.06	0.66	0.68
**Margin** ^**†**^	0.85	0.53	0.67	0.84	0.57	0.64

M is the mean group error, Σ is the inter-patient uncertainty and σ is the inter-fraction uncertainty. The sigma values are used to calculate the needed CTV-PTV margin using the van Herk formalism.

^†^ Margin not including patient 7, see discussion section.

**Figure 2:**
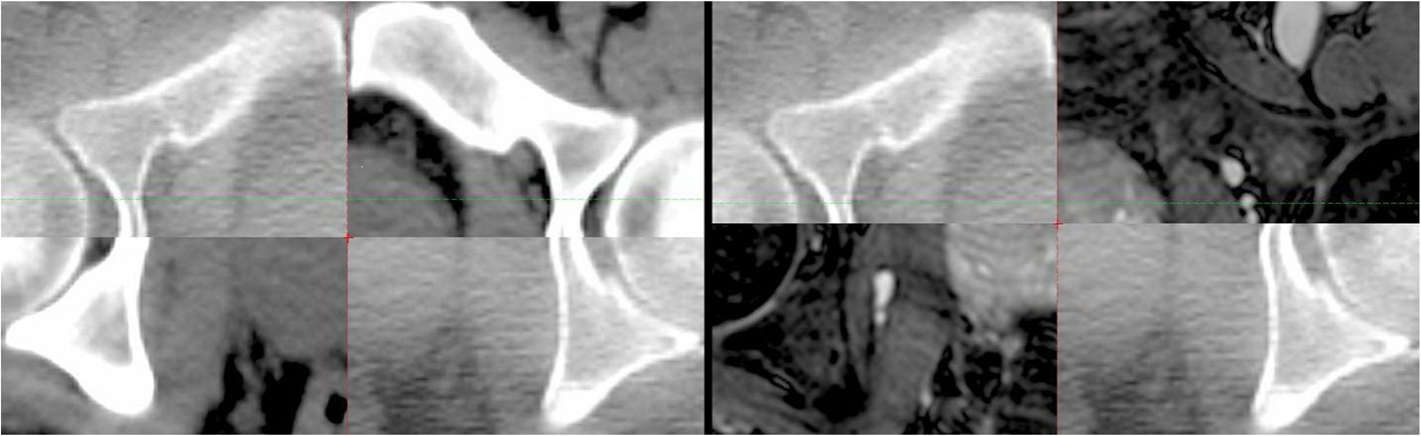
**Registration overlays of CBCT with either CT (on the left) or MRI (on the right).**

The margins for the CBCT-CT registration were 0.87 cm (AP), 0.64 cm (SI) and 0.69 cm (LR) compared to the CBCT-MRI registration values of 1.06 cm (AP), 0.66 cm (SI) and 0.68 cm (LR). These margin calculations show that CBCT-MRI registration is within two millimeters of those between CBCT-CT. The largest margin difference is in the AP direction. This is due to bowel preparation issues at time of MRI simulation. This issue is expanded on in the discussion section. Imaging modality did not impact daily localization. MRI performs as well as CT as a reference image for registration. Figure 2 shows the capability of T1-MRI for providing an image reference set for daily CBCT by comparing the respective CT/MRI registration to the same CBCT slice. Some CBCT-MRI registrations were performed multiple times in order to measure the reproducibility, with the registration uncertainty (standard deviation) being 0.08, 0.09 and 0.07 cm in the AP, SI and LR directions, respectively.

## Discussion

We found clear visual improvement in anatomical delineation from MRI as seen by the agreement of our volume differences compared to the literature [25]. The penile bulb is often difficult to discern on CT. The difference, both spatial and volumetric, between the MRI volume and the CT volume are stark. Consequently, if using only CT, we would not be able to offer reduction of the collateral radiation damage to these structures during radiation planning and delivery, which may alter the quality of life of patients treated with radiation.

A novel aspect of our study was using MRI as the reference dataset for 3D-3D CBCT-MRI matching for image-guided RT. Such data has not been previously reported for the prostate. Since patients underwent CT and MRI simulation, we could compare the shifts and calculated planning margins obtained with the both datasets. Looking closely at each patient, differences in shifts between MRI and CT registration (Table 4) are consistent overall for each modality. The only registration difference greater than 3 mm between CT and MRI is for patient 7 in the AP direction. This is due to non-compliance of bowel preparation at the MRI and CT simulations. Due to non-compliance, the prostate position relative to bony anatomy changed significantly. Consequently this dramatically impacts registering the CT and MRI images, particularly in the AP direction, which resulted in systematic differences (>6 mm) between MRI and CT-generated shifts. If we exclude this outlier data from the analysis, the margins are nearly identical between MR and CT, as observed in Table 4. A previous 3D-3D CBCT-MRI matching study performed by Buhl et al. [26] reported on CBCT shifts compared to reference MRI images of the brain and showed mean and standard deviation values of 0.8 ± 0.6 mm, 1.5 ± 1.2 mm and 1.2 ± 1.2 mm differences in the AP, SI and LR directions, respectively. These compare very well to our patient cohort mean and standard deviation values of 1.5 ± 2.5 mm in the AP direction, 0.7 ± 1.9 mm in the SI direction and −0.1 ± 1.4 mm in the LR direction.

One disadvantage of the study was that we could not simulate both MRI and CT during the same appointment session. Since patients had to return for another image acquisition there were instances where rectal/bladder filling was inconsistent between sessions. This created a source of uncertainty during the image registration process since changes in bladder and rectum filling could deform other organs such as the seminal vesicles or prostate. In the clinical implementation of a MRI-only workflow, this source of error would be mitigated.

**Figure 3:**
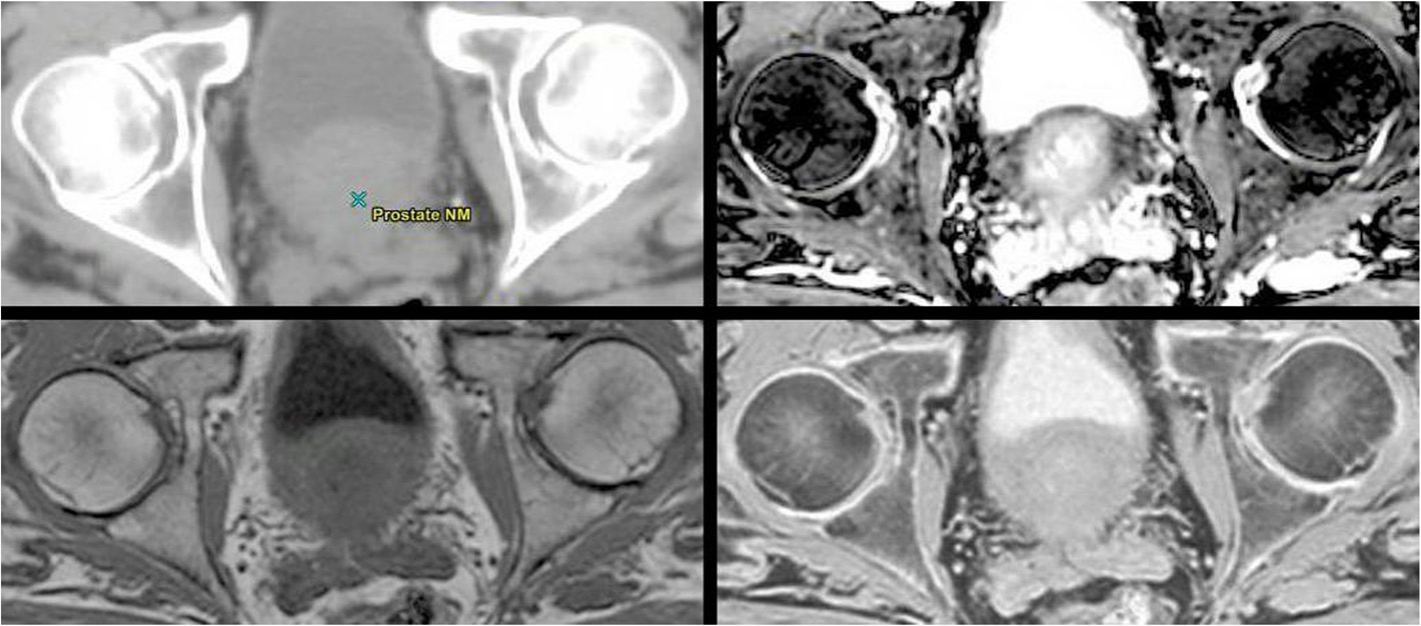
**Different imaging sequences comparing bone boundaries.** Detailed Legend: Imaging sequence from top left going clockwise. CT, bTFE, Inverted T1, T1. Notice that boundary of the femoral heads and other bony tissue shows up as a region of no signal in the bTFE and T1 image. On the CT and the inverted T1 image, this edge is readily identifiable.

During our study, we spent a great deal of effort outlining the femoral heads and pelvic bones. MRI simulation and treatment planning relies on correctly delineating all structures since bulk electron densities are assigned. We found normal T1 and T2 image acquisitions provided substandard image quality to properly delineate these bones. bTFE acquisition provided adequate image quality at the expense of additional scan time. Inverting the T1 image grayscale gave a clearer outline of the edge of the femoral heads and other bony structures compared to T1 and T2 images. Image comparisons can be seen in Figure 3. Future utilization of ultra-fast (UTE) [22] and water/fat separating multi-echo (mDIXON) [27] sequences are likely necessary for proper identification of bony anatomy. Image registration to the daily CBCT was found to be straightforward and uncomplicated. Aligning the capsule edge, seminal vesicles, rectum interface and the femoral heads was no more difficult than with a CT reference image.

MRI simulation is not without limitations. The first consideration with MRI simulation is whether to use open or closed bore systems. Closed bore systems allow for greater magnet strength, useful for MRI spectroscopy and reducing overall scan time. Open-bore systems consist of 2 horizontal magnets. This open design allows for greater patient comfort and allows simulation therapists increased access to patients, just as in CT simulation. However, as open-bore systems tend to use lower strength magnets which can adversely impact the signal-to-noise ratio. Our open-MRI system uses rigid radiofrequency (RF) coils with integrated solenoid technology and receiver elements perpendicular to the body’s long axis. These are mounted to the patient table to improve the signal-to-noise ratio. Using these coils allows our 1.0 T vertical system to maximize image quality. Enders et al. [28] found out that in a blinded randomized study evaluating vertical and horizontal field image quality, closed-bore systems had better image quality, though open-bore systems still had an average image quality grade of ‘moderate’, which is acceptable in non-diagnostic environments. The last consideration in bore style is the need to accommodate immobilization devices that are increasingly utilized in radiation oncology departments in order to fixate patients and minimize patient motion during simulation and subsequent treatment. Open-bore systems are more accommodating to a wider arrange of immobilization devices.

Magnetic field distortions may cause geometric aberrations that can result in inaccurate identification of the patient’s skin contour, a crucial component to radiation treatment planning [16,17]. It is important that geometric distortions of each MRI scanner used for simulation be characterized so that associated uncertainties across the FOV are understood [29,30]. Geometric distortions are classified as system distortions or object-related distortions. Geometric distortion is evaluated at our institution on a daily basis for all three axes using a vendor provided 2-dimensional geometric distortion phantom with a field of view (FOV) encompassing imaging sequence (~35 cm x 40 cm). The difference in center of mass position between the acquired image and the ideal position map is measured. Deviations are displayed as isocurves that delineate 2 mm distortion within the primary FOV up to 6 mm of distortion at the FOV edge. The plot is visually inspected to insure the 2 mm isocurve does not advance past the peripheral imaging region. Object-related distortions are another concern and cannot be evaluated with the use of a phantom. Multiple proposals exist for assessing and minimizing object-related distortion [18,19,31-33], but the issue remains uncorrected in clinical settings. Distortions due to tissue susceptibility scale almost linearly with the B_0_ field strength, and can be considered to be minimal (<0.5 mm) at a field strength of 1.0 T [31]. Relative to CT, uncorrected MRI geometric uncertainties can degrade contouring accuracy while the enhanced soft-tissue contrast improves contouring precision.

Electron density information will always be necessary for accurate dose calculation. For this study only two densities were assigned, but in other areas of the body where air pockets or other tissue densities exist, this could be a bottleneck for treatment planning efficiency without intelligent autosegmentation tools. For MRI-only simulation, we will need to quickly and accurately determine a tissue’s electron density. One possibility is to create so-called “synthetic CTs”, which are currently being researched to infer electron density information directly from MRI [21,34]. For metallic implants such as hip prostheses, CT can use scatter correction algorithms to correct for this presence [35], whereas MRI is compromised by the metal artifact and could represent a contraindication for MRI-only simulation. Additional safety concerns need to be thoroughly investigated and due diligence performed; any medical product introduced into a high magnetic field environment must be properly vetted. Examples include implanted medical devices and infusion catheters. Our clinic found it necessary to complete MRI screening forms with the patients three times prior to simulation in order to guarantee that incompatible materials do not enter the magnetic field. Patients that are non-compliant in this regard cannot currently be simulated in a MRI-only simulation workflow.

## Conclusion

This study confirms that MRI has the potential to be a primary imaging modality for treatment planning simulation, dose calculations, and daily image localization. Readily available radiation oncology directed MR simulation can increase the population of patients that take advantage of more precise tumor delineation given the improved soft tissue contrast with MRI. Consequently it is possible to develop more accurate treatment plans, which have the potential to positively influence patient outcomes. Ultimately this hypothesis needs to be tested, and in this regard, we are currently performing a prospective clinical trial to correlate patient outcomes (toxicities and tumor control) with dose distributions generated using MRI-defined targets.

### Consent

Written informed consent was obtained from the patients for the publication of this report and any accompanying images.
